# Comparing the efficacy and neuroinflammatory potential of three anti-abeta antibodies

**DOI:** 10.1007/s00401-015-1484-2

**Published:** 2015-10-03

**Authors:** James P. Fuller, Jeffrey B. Stavenhagen, Søren Christensen, Fredrik Kartberg, Martin J. Glennie, Jessica L. Teeling

**Affiliations:** Centre for Biological Sciences, University of Southampton, Southampton, UK; Lundbeck A/S, Copenhagen, Denmark; Cancer Sciences, University of Southampton, Southampton, UK

**Keywords:** Immunotherapy, Alzheimer’s disease, Bapineuzumab, Gantenerumab, Crenezumab, Inflammation

## Abstract

Immunotherapy is a promising strategy for the treatment of Alzheimer’s disease (AD). Antibodies directed against Amyloid Beta (Aβ) are able to successfully clear plaques and reverse cognitive deficits in mouse models. Excitement towards this approach has been tempered by high profile failures in the clinic, one key issue has been the development of inflammatory side effects in the brain (ARIAs). New antibodies are entering the clinic for Alzheimer’s disease; therefore, it is important to learn all we can from the current generation. In this study, we directly compared 3 clinical candidates in the same pre-clinical model, with the same effector function, for their ability to clear plaques and induce inflammation in the brain. We produced murine versions of the antibodies: Bapineuzumab (3D6), Crenezumab (mC2) and Gantenerumab (*ch*Gantenerumab) with an IgG2a constant region. 18-month transgenic APP mice (Tg2576) were injected bilaterally into the hippocampus with 2 µg of each antibody or control. After 7 days, the mice tissue was analysed for clearance of plaques and neuroinflammation by histology and biochemical analysis. 3D6 was the best binder to plaques and in vitro, whilst mC2 bound the least strongly. This translated into 3D6 effectively clearing plaques and reducing the levels of insoluble Aβ, whilst *ch*Gantenerumab and mC2 did not. 3D6 caused a significant increase in the levels of pro-inflammatory cytokines IL-1β and TNFα, and an associated increase in microglial expression of CD11B and CD68. *ch*Gantenerumab increased pro-inflammatory cytokines and microglial activation, but minimal changes in CD68, as an indicator of phagocytosis. Injection of mC2 did not cause any significant inflammatory changes. Our results demonstrate that the ability of an antibody to clear plaques and induce inflammation is dependent on the epitope and affinity of the antibody.

## Introduction

One neuropathological hallmark of Alzheimer’s disease (AD) is the accumulation of amyloid β (Aβ) as extra-cellular deposits. Data from both familial and sporadic forms of AD led to the hypothesis that Aβ is central to the pathogenesis of AD [15]. As a result there has been a great effort to develop drugs that will reduce the production of Aβ or clear the deposits. One approach has been to immunise against Aβ, either by active immunisation against Aβ or by passive immunisation with anti-Aβ antibodies. Active immunisation of transgenic APP mice with Aβ clears plaques and reverses memory deficits [25], but active immunotherapy in AD patients is associated with severe inflammatory side effects [20]. Passive immunotherapy with monoclonal anti-Aβ antibodies also reduces amyloid deposition and reverses cognitive deficiencies in transgenic APP mice [2, 9, 32]. Due to this success in experimental models, several monoclonal antibodies reached clinical trials for the treatment of AD, but excitement towards this approach has been tempered by a number of high profile failures to deliver disease-modifying effects. Bapineuzumab and Solanezumab both failed in phase III, and a phase III trial for Gantenerumab was abandoned after a futility analysis [12, 23]. With more antibodies for AD entering the clinic, including Aducanumab, which has shown promising effects in Phase I, it is imperative to learn from the first-generation antibody therapies to inform the development of new and improved clinical candidates. Bapineuzumab, a humanised IgG1, which recognised the N terminus of Aβ cleared plaques from the brains of patients in a phase II trial, however, it also caused oedema and micro-haemorrhage of the cerebral vasculature [22, 24]. These side effects limited the top dose in phase III trials, which potentially contributed to the lack of efficacy [23]. These side effects have also been observed with Gantenerumab (hIgG1, N terminus conformational epitope). Pre-clinical studies have suggested that side effects could be due to inflammation caused by Fc effector function of the therapeutic antibodies, as de-glycosylation can prevent vascular damage in vivo [6, 13, 29]. With this in mind, Crenezumab (hIgG4, mid-domain epitope) was generated with a human IgG4 constant region to modify Fc effector function and reduce vascular side effects [1].

The efficacy to clear plaques and the potential to induce side effects is difficult to predict due to inconsistent use of experimental models, epitope specificity of the antibody tested (N, mid or C terminus) or antibody subclass. In this study, we set out to compare three antibodies which have been in phase III clinical trials for AD: Crenezumab, Bapineuzumab and Gantenerumab. Bapineuzumab is a human IgG1 antibody, which binds to the N terminus of Aβ (AA 1–5), and can recognise both soluble and insoluble Aβ [23]. Gantenerumab is also a human IgG1 antibody which recognises a conformational epitope, making contact with the n terminus and the mid-domain of Aβ, reportedly binding more strongly to more aggregated forms compared to soluble Aβ [3]. Crenezumab is a human IgG4 antibody which recognises a mid-domain epitope (AA 16-24), and binds to all forms of Aβ [1]. We produced murine versions of Crenezumab (mC2), Bapineuzumab (3D6) and a mouse/human chimeric version of Gantenerumab (*ch*Gantenerumab) all with an IgG2a constant regions. We compared these antibodies for their ability to clear Aβ plaques and to induce neuroinflammation following intracerebral injection into transgenic APP mice (Tg2576). Murine IgG2a subclass was selected as this subclass is similar to hIgG1 in terms of FcγR binding, and hIgG1 has been the most commonly used subclass for anti-Aβ immunotherapy. Here, we show that the affinity and epitope specificity are important for the efficient removal of Aβ from the CNS, but also for the induction of a neuroinflammatory response.

## Methods

### Recombinant antibody production

Antibody primary sequences for the variable domains of 3D6, Gantenerumab and mC2 along with sequences for the constant regions of the mouse IgG2a heavy chain and mouse kappa light chain were identified from the public domain. Synthetic genes for the variable light chain-murine Kappa constant domain and variable heavy chain-mIgG2a constant domain were sub-cloned into the pTT5 plasmid for transient gene expression (Geneart). Primary structures of the expressed antibodies are shown in Table 1. Transfection of heavy chain and light chain expression vectors was performed in HEK293 6E cells using PEIpro (Polyplus) as a transfection reagent. The HEK293 6E expression system including the pTT5 vector is licensed from the National Research Council of Canada. Transfected cells were cultured until the viability had dropped to around 50 % and culture media harvested by centrifugation and ultrafiltration. Antibodies were purified from clarified culture media by protein-G Sepharose (GE healthcare) affinity chromatography followed by online desalting into PBS (Invitrogen) using ÄKTA express system and standard protocols for monoclonal antibody purification. Finally, the antibodies were sterile filtered using 0.2-µm filters (Millipore) and kept at 4 °C. Purified antibodies were analysed for purity by SDS-PAGE and Bioanalyzer 2000, aggregation by uplc-sec (Waters), concentration by Pierce BCA kit (ThermoFisher) and endotoxin by endosafe PTS (all <0.025 EU/mg, Charles River). Confirmation of the primary structure was done by analysing the intact mass of the heavy chain and light chain by RP-uplc (C4 column in 0.1 %FA; ACN gradient) followed by ESI–MS on a QTOF instrument (Waters XEVO QTOF). Multicharged spectra were processed using the MaxEnt 1 deconvolution algorithm.

**Table 1:** Sequences of antibodies used in study

Antibody	Heavy chain variable domain sequence	Light chain variable domain sequence	References
3D6 (Murine)	EVKLVESGGGLVKPGASLKLSCAASGFTFSNYGMSWVRQNSDKRLEWVASIRSGGGRTYYSDNVKGRFTISRENAKNTLYLQMSSLKSEDTALYYCVRYDHYSGSSDYWGQGTTVTVSS	YVVMTQTPLTLSVTIGQPASISCKSSQSLLDSDGKTYLNWLLQRPGQSPKRLIYLVSKLDSGVPDRFTGSGSGTDFTLKISRIEAEDLGLYYCWQGTHFPRTFGGGTKLEIK	US Patent No. 7,790,856B2
Gantenerumab (Human)	MWTLVSWVALTAGLVAGQVELVESGGGLVQPGGSLRLSCAASGFTFSSYAMSWVRQAPGKGLEWVSAINASGTRTYYADSVKGRFTISRDNSKNTLYLQMNSLRAEDTAVYYCARGKGNTHKPYGYVRYFDVWGQGTLVTVSS	MWTLVSWVALTAGLVAGDIVLTQSPATLSLSPGERATLSCRASQSVSSSYLAWYQQKPGQAPRLLIYGASSRATGVPARFSGSGSGTDFTLTISSLEPEDFATYYCLQIYNMPITFGQGTKVEIK	EMBL database ID: CHEMBL1743025
mC2 (Murine)	EVQLVESGGGLVQPGGSLKLSCAASGFTFSSYGMSWVRQTPDKRLELVASINSNGGSTYYPDSVKGRFTISRDNAKNTLYLQMSSLKSEDTAMYYCASGDYWGQGSTLTVSS	DVVMTQTPLSLPVSLGDQASISCRSSQSLVYSNGDTYLHWYLQKPGQSPKLLIYKVSNRFSGVPDRFSGSGSGTDFTLKISRVEAEDLGVYFCSQSTHVPWTFGGGTKLEIK	WO 2008011348 A2 WO 2007068412 A2

	Mouse IgG2A Constant domain	Mouse Kappa chain constant domain	Uniprot ID
	AKTTAPSVYPLAPVCGDTTGSSVTLGCLVKGYFPEPVTLTWNSGSLSSGVHTFPAVLQSDLYTLSSSVTVTSSTWPSQSITCNVAHPASSTKVDKKIEPRGPTIKPCPPCKCPAPNLLGGPSVFIFPPKIKDVLMISLSPIVTCVVVDVSEDDPDVQISWFVNNVEVHTAQTQTHREDYNSTLRVVSALPIQHQDWMSGKEFKCKVNNKDLPAPIERTISKPKGSVRAPQVYVLPPPEEEMTKKQVTLTCMVTDFMPEDIYVEWTNNGKTELNYKNTEPVLDSDGSYFMYSKLRVEKKNWVERNSYSCSVVHEGLHNHHTTKSFSRTPGK	RADAAPTVSIFPPSSEQLTSGGASVVCFLNNFYPKDINVKWKIDGSERQNGVLNSWTDQDSKDSTYSMSSTLTLTKDEYERHNSYTCEATHKTSTSPIVKSFNRNEC	P01863

Amino acid sequences of variable regions for antibodies: 3D6, Gantenerumab and mC2 and sequences for the constant heavy domain of mouse IgG2a and the mouse kappa domain. All sequences were obtained from the public domain

### Abeta binding assay

Binding characteristics of the recombinant antibodies to Aβ peptide was assessed as follows: 96-well Maxisorp plates (Nunc, Denmark) were coated with 100 µl of 0.1 µg/ml Aβ _1–40_ peptide (America Peptide, Sunnyvale, CA) in bicarbonate buffer (pH 9.6) overnight at room temperature. Plates were washed 5 times with PBS/0.1 % Tween and recombinant anti-Aβ antibodies were serially diluted in 100 µl of assay buffer (PBS/1 % BSA) and incubated for 1 h at room temperature. After washing plates 5 times with PBS/0.1 % tween, antibody binding was detected by the addition of 100 µl of using horse anti-mouse IgG conjugated to biotin (vector) diluted 1:200 in assay buffer. Plates were washed 5 times before addition of 100 µl streptavidin poly-HRP (1:10,000 in assay buffer, Sanquin, the Netherlands). Bound antibody was detected using tetramethylbenzidine (TMB) (Sigma) as a substrate. The substrate solution was made by dissolving 1 TMB tablet in 1 ml of DMSO, which was made up to 10 ml in phospho-citrate buffer (Sigma), 2 µl of 30 % H_2_O_2_ (Sigma) was added to the solution directly before addition to the plate. 100 µl of the substrate solution was added to each well and allowed to develop. The reaction was stopped by the addition of 50 µl H_2_SO_4_ to each well, and absorbance was measured at 450 nm using a Fluostar Optima™ spectrophotometer.

### Cell culture and effector function assay

The mouse macrophage cell line RAW 264.7 was cultured in DMEM media (Gibco) supplemented with 10 % heat inactivated foetal calf serum (FCS, Sigma) and 2 mM glutamine (Sigma). Cells were incubated at 37 °C with 5 % CO_2_. Effector function of the recombinant antibodies was measured in vitro by immobilising antibodies (100 µl/well, 5 µg/ml antibody in pH 9.6 bicarbonate buffer) to U.V. sterilised 96 well ELISA plates (Nunc, Maxisorp Flat bottom). Unbound antibodies were removed by washing the plates with DMEM, plates were then blocked with cell culture medium containing 10 % FCS for 1 h, then 4 × 10^4^ RAW cells per well were added in DMEM media containing 100 units per ml of murine IFN-γ (Peprotech). The cells were cultured for 24 h and supernatant removed to measure the levels of TNFα by ELISA.

### TNFα ELISA

TNFα levels were measured in cell culture supernatant using TNFα duoset™ ELISA (RnD systems), according the manufacturer’s instructions, with minor modification. Briefly, capture antibodies were coated onto maxisorp plates in 100 µl 0.1 M bicarbonate buffer pH 9.4 overnight at room temperature. The plates were washed 5 times in PBS 0.05 % Tween. Cell culture supernatant or TNFα standard serially diluted in assay buffer (PBS/1 % BSA) was added and plates incubated for 2 h on a shaking table. After washing the plates, the secondary biotinylated antibody was added incubated for a further 2 h, before the addition of poly-HRP (1:10000 in PBS/1 %BSA, Sanquin, the Netherlands). This assay was developed and measured in the same way as the Aβ binding ELISA.

### Animals and stereotaxic surgery

Female Tg2576 transgenic mice possessing a copy of human APP with the Swedish double mutate on (KM670/671NL) were obtained from Taconic and aged to 18 months. Mice were kept in 12 h light–dark cycles with free access to food and water. Mice were randomised into 4 groups (3D6, mC2, Gantenerumab or irrelevant mIgG2a control (*n* = 6/7 per treatment group). Mice were weighed and anaesthetised using sevoflurane, and secured onto a stereotaxic frame. A mid-sagittal incision was made exposing the skull, and two burr holes were made at coordinates (−2.0, +1.7) and (−2.0, −1.7). A fire pulled glass capillary with a diameter of <50 μm (Sigma) was inserted −1.4 mm into the brain and 2µl of antibody (1 mg/ml) was slowly injected bilaterally. This method allows accurate injection of 2 µg of mAb with minimal mechanical damage. Sutures were used to close scalp and mice were left to recover in a thermo-regulated environment. All procedures were carried out in accordance with Danish law.

### Tissue processing

Seven days after the injections the mice were terminally anaesthetised using avertin, and transcardially perfused with heparinised saline. The brain was removed and cut to two hemispheres. The left hemisphere was further dissected, and a hippocampal punch was taken as described before [16] and frozen in isopentane over dry ice. The right hemisphere was mounted in optimal cutting temperature medium (OCT, Sakura Finetek, Thatcham, UK) for immunohistochemistry.

### Immunohistochemistry

Brains were cut into 10 micron sections using a cryostat. A series of sections through the hippocampus was taken, and the site of injection was located by staining for CD11B; this allowed identification of the antibody-injected brain region to use for further immunohistochemistry. Immunohistochemistry was performed as described before [27] using CD11B (5C6, 1:500, Serotec), CD68 (FA11, 1:500, Serotec) and Aβ (3D6, 1:1000, in house), FITC sheep anti-mouse F(ab)2 (1:500, Sigma), horse anti-mouse biotinylated (1:250, Vector) and rabbit anti rat biotinylated (1:250, Vector).

### Congo red staining

Ten micron sections were dried at 37 °C for 30 min, and fixed in 4 °C ethanol for 15 min before washing in three changes of PBS. Sections were stained for 15 min in 0.3 % Congo red (Sigma) in 80 % ethanol containing 0.01 % NaOH (Sigma). Sections were de-stained by dipping five times in 50 % ethanol containing 0.02 % NaOH, and transferred into PBS. Slides were mounted using Pro-long gold™ anti-fade reagent (Invitrogen) containing the counterstain DAPI.

### Aβ staining in human tissue

The postmortem tissue of the AD cases was provided by BRAIN UK under the ethics approval obtained from the National Research Ethics Committee South Central Hampshire B (REC reference 14/SC/0098). Formalin-fixed paraffin-embedded brain tissue was cut into 5 µm sections on a microtome. Tissue was dewaxed in clearene (Surgipath), and rehydrated in graded alcohols. Endogenous peroxidase activity was quenched by incubation of tissue with 3 % H_2_O_2_ in methanol for 10 min, before 3 washes with tris-buffered saline (TBS). Sections were then either incubated for 30 min in 80 % formic acid (Fisher) or in TBS. Sections were blocked for 30 min with medium containing DMEM, FCS BSA. Anti-Aβ antibodies were added at a concentration of 1:1000 in TBS and incubated at 4 °C overnight. Sections were then washed in 3 rinses of TBS before addition of biotinylated rabbit anti-mouse IgG secondary (Abcam, 1:600) and incubated for 1 h. Bound antibody was detected by incubation for 30 min with ABC system (Vector), and washed 3 times in TBS, before developing with diaminobenzidine solution (DAB, Vector). Slides were dehydrated in graded alcohols into clearene, before mounting with pertex™ (Cellpath).

### Quantification of immunohistochemistry

Light microscopy (DAB) and fluorescent images were taken on a Leica DM5000 microscope using QWIN or LAS software. The quantification of DAB images was performed using the freeware—ImageJ. Images taken with a 20× objective were de-convoluted using HDAB RGB values, which split images into haematoxylin and DAB channels. The DAB staining was turned into a binary image, and the percentage area above a marker-specific threshold was measured. To quantify congophilic plaques, the number of Congo red-positive deposits was counted in the hippocampus. The number of plaques was then normalised to hippocampal area. During all quantification, the slides were blinded to prevent bias.

### MSD measurement of Aβ and cytokines

Protein was extracted from hippocampal punches in 2 stages, as described previously [26]. First, hippocampal tissue was placed in a buffer containing: 150 mM NaCL, 25 mM TRIS 1 % triton x-100 and complete mini protease inhibitors (Roche). The tissue was mechanically homogenised and the homogenate was centrifuged at 20,000*g* at 4 °C for 1 h to remove insoluble material. The supernatant from this fraction was used to measure pro-inflammatory cytokine levels and the triton soluble Aβ fraction using multiplex MSD technology. The pellet was re-suspended in 70 % formic acid and incubated for 15 min to solubilise aggregated forms of Aβ, this solution was neutralised in 20 volumes of pH8 TRIS, and centrifuged at 20,000*g* for 1 h and the supernatant used to measure the formic acid soluble Aβ fraction. Total protein levels were measured by BCA protein assay (Pierce), and final levels were expressed as pg/mg protein. The multiplex Aβ assay c-terminal antibodies to capture specific Aβ peptides, and uses the antibody 6E10 (AA 1-16) for detection.

### Statistical analysis

All data are expressed as mean ± standard deviation. Statistical analysis was performed and figures created using Graphpad Prism software. Data were first analysed for normal distribution; normally distributed data were analysed by one-way ANOVA and then treatment groups were compared using TUKEY post hoc analysis. Non-parametric data were analysed using non-parametric equivalents.

## Results

### Antibody characterisation

Three murine anti-Aβ antibodies: 3D6, mC2 and *ch*Gantenerumab were generated recombinantly from the variable domain amino acid sequences. All antibodies were produced with the same mouse IgG2a constant regions, to ensure they had the same effector function. The antibodies were purified and free from endotoxin and IgG aggregates prior to use in experiments. 3D6 and mC2 were the original parent antibodies of Bapineuzumab and Crenezumab.

**Fig. 1:**
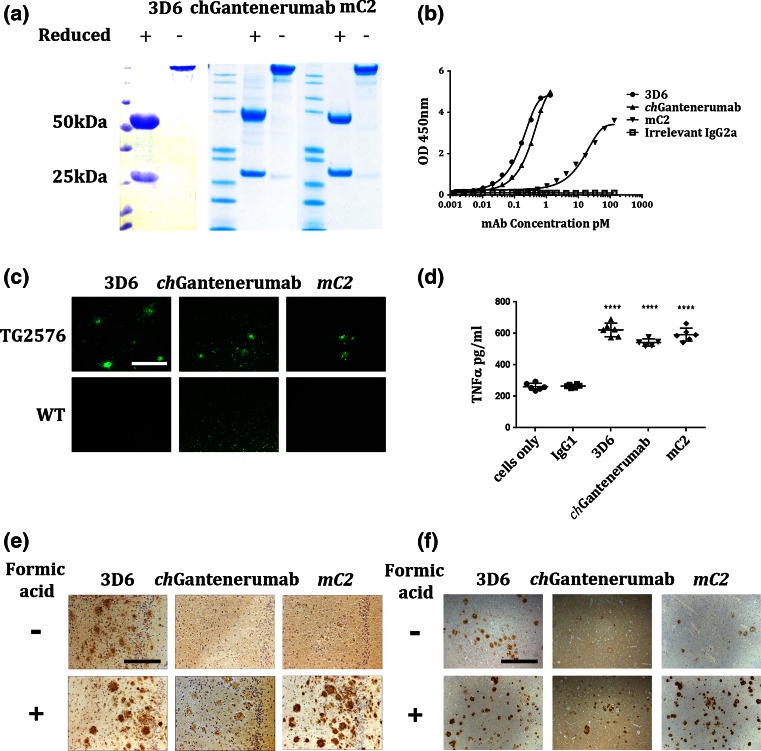
In vitro binding and effector function of 3D6 chGantenerumab and mC2. **a** Purified recombinant antibodies were separated by reduced and non-reduced polyacrylamide electrophoresis. Non-reduced antibodies run as a single band, reduced antibodies break into heavy and light chain fragments (50, 25 kDa respectively). **b** Binding of anti-Aβ antibodies to immobilised Aβ _1–40_. 3D6 and chGantenerumab both bind with relatively high affinity (EC50 3D6 = 0.17 pM; EC50 chGantenerumab = 0.34 pM) mC2 bound with lower relative affinity (EC50 mC2 = 17.4 pM). **c** Binding of 3D6, *ch*Gantenerumab and mC2 to Tg2576 (APP) and wild-type (WT) brain sections. **d** TNFα levels produced by RAW264.7 cells in response to immobilised Aβ antibodies or IgG1 control. Data analysed by one-way ANOVA and Tukey post hoc test, and expressed as mean pg/ml supernatant ± standard deviation (SD, *n* = 6). Data is representative of 3 independent experiments (****p < 0.0001). **e** Binding of antibodies to formalin-fixed tissue from Tg2576 mice, with and without formic acid antigenic retrieval. **f** Binding of antibodies to human AD brain tissue, with and without formic acid antigen retrieval

First, the specificity and relative affinity to Aβ were tested by measuring binding to immobilised Aβ 1–40 peptide. Figure 1b shows that both 3D6 and bind to recombinant peptide with relative high affinity (EC50 3D6 = 0.17 pM; EC50 *ch*Gantenerumab = 0.34 pM), however, 100-fold higher levels of mC2 were required to reach half maximal binding (EC50 mC2 = 17.4 pM), suggesting significantly lower affinity to immobilised Aβ. Antibodies were then tested for binding to Aβ plaques in brain sections from Tg2576 mice, and to better mimic in vivo binding conditions tissue sections were not subjected to any antigen retrieval before immuno-staining. 3D6 bound plaques in tissue obtained from Tg2576, while no binding was observed in wild-type mice. mC2 also bound but fewer plaques were labelled (Fig. 1c). *ch*Gantenerumab labelled plaques, however, it also appeared to bind to neurons in both Tg2576 and wild-type mice. All antibodies were produced as IgG2a isotype, using the same constant region, and therefore should all have the same ability to bind and activate FcγRs. We then tested the ability of these antibodies to bind to plaques in Tg2576 tissue sections with and without formic acid antigenic retrieval (Fig. 1e). Formic acid treatment breaks down aggregated Aβ into more soluble species. We found that 3D6 was able to bind to plaques without any antigen retrieval, but mC2 and c*h*Gantenerumab could not. After formic acid treatment, mC2 labelled plaques very well and *ch*Gantenerumab labelled them faintly. The conformation of Aβ in Tg2576 mice and human AD cases may be different, and this has previously been reported to affect target engagement of anti-Aβ antibodies [28]. To characterise the ability of antibodies to bind to Aβ from human cases, brain sections from AD cases were stained with and without formic acid antigenic retrieval (Fig. 1f). The results were comparable to Tg2576 tissue, with 3D6 able to bind plaques without antigenic retrieval, but staining improved after formic acid treatment. The antibody mC2 bound poorly without formic acid but labelled plaques well after treatment. *ch*Gantenerumab again showed background staining, but plaque binding was evident after formic acid treatment. Using an FcγR crosslinking assay, we tested the ability of each antibody to activate macrophages in vitro. All IgG2a anti-Aβ antibodies stimulate secretion of the cytokine TNFα compared to cell only controls, while mouse IgG1, a subclass which has lower affinity for activating FcγR, fails to induce TNFα secretion (Fig. 1d). In summary, 3D6 binds plaques and recombinant Aβ 1–40 peptide with high affinity and specificity, mC2 selectively binds Aβ but with lower affinity, while *ch*Gantenerumab binds Aβ 1–40 peptide with high affinity, in situ binding indicates non-specific binding to neurons. All antibodies have the comparable ability to activate macrophages through FcγRs.

### Clearance of Aβ

**Fig. 2:**
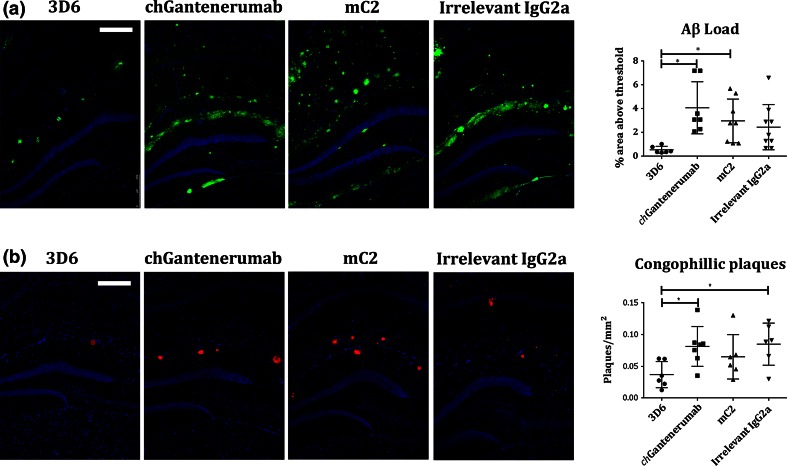
Anti-Aβ immunohistochemistry and congophilic plaque load. **a** Tissue sections from Tg2576 mice injected with antibodies were immuno-stained for Aβ, and the percentage staining area above threshold was measured. Data were analysed by Kruskal–Wallis and Dunn’s post hoc test and expressed as mean ± SD (*n* = 6/7). 3D6 significantly reduced Aβ load compared to mC2 and *ch*Gantenerumab (*p* = 0.0265 and *p* = 0.0013, respectively). Images taken with a ×10 objective, *scale bar* 250 µm. **b** To test for clearance of congophilic plaques, brain sections from were stained with Congo red. The numbers of congophilic plaques were counted and normalised to hippocampal area and expressed as congophilic plaques/mm^2^. Data were analysed by one-way ANOVA and Tukey post hoc test and expressed as mean ± SD (*n* = 6/7). 3D6 significantly reduced the number of congophilic plaques compared to irrelevant IgG2a and chGantenerumab (*p* = 0.0265 and *p* = 0.0178, respectively). Images taken with a ×10 objective, *scale bar* 250 µm

**Fig. 3:**
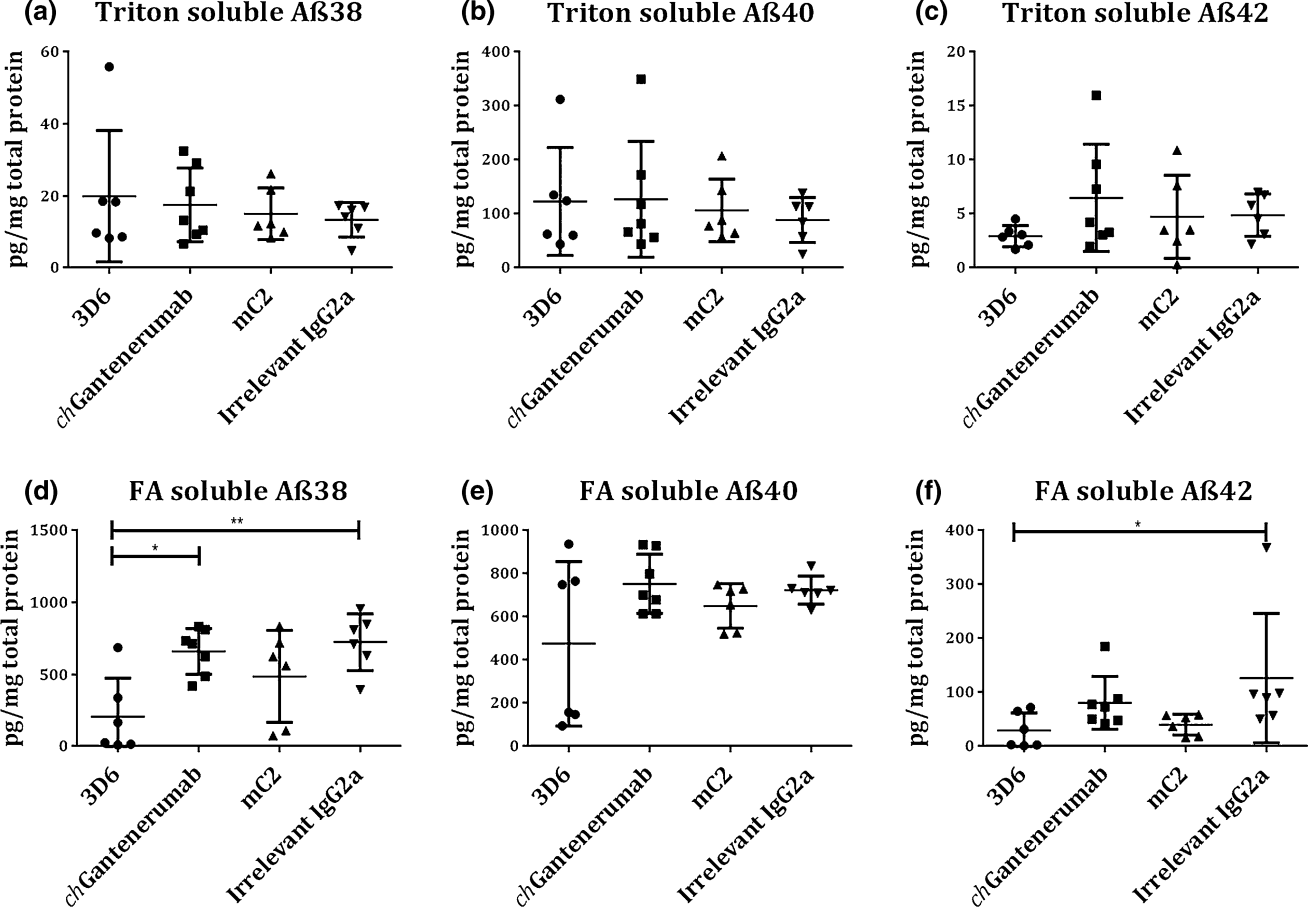
Soluble and insoluble Aβ levels measured by multiplex ELISA. The levels of 3 different Aβ peptides were measured by multiplex ELISA from two different fractions of brain homogenate: triton soluble and formic acid soluble. Aβ concentration was normalised to total protein levels and expressed as pg/mg total protein. **a–c** Levels of triton soluble Aβ38, Aβ40 and Aβ42, respectively. **d–f** Levels of formic acid soluble Aβ38, Aβ40 and Aβ42, respectively. With the exception of **e**, data were analysed by one-way ANOVA with Tukey post hoc test **e** was analysed by Kruskal–wallis and Dunn’s post hoc. Data is expressed as mean ± SD (*n* = 6/7). 3D6 significantly reduced the amount of formic acid soluble Aβ38 compared to *ch*Gantenerumab and irrelevant IgG2a control (*p* = 0.0168 and *p* = 0.0073, respectively) and reduced levels of formic acid soluble Aβ 42 compared to irrelevant IgG2a (Fig. 3f, *p* = 0.041)

Next, we tested the ability of the anti-Aβ antibodies to clear Aβ in vivo by injecting 2 µg of each antibody into the hippocampus of 18-month-old Tg2576 mice. Clearance of Aβ after intracerebral injection of antibody into Tg2576 mice was quantified by Aβ immuno-staining. Injection of 3D6 significantly reduced Aβ plaque load in comparison to *ch*Gantenerumab and mC2 (Fig. 2a, *p* = 0.0013 and *p* = 0.026, respectively). Due to the potential of antigenic masking by injected antibodies, Congo red staining was used to detect the clearance of congophilic deposits after antibody injection. 3D6 significantly reduced the number of Congo red-positive plaques in the hippocampus compared to irrelevant IgG2a and *ch*Gantenerumab injected animals (Fig. 2b, *p* = 0.0265 and *p* = 0.0178, respectively). The results were confirmed by measuring the levels Aβ38, Aβ40 and Aβ42 in brain homogenate. Diffuse (triton soluble) Aβ levels were not affected 7 days post-injection. Supporting the decreased immuno-staining, 3D6 significantly reduced the amount of aggregated (formic acid soluble) Aβ38 compared to Gantenerumab or irrelevant IgG2a injection (Fig. 3d, *p* = 0.0168 and *p* = 0.0073, respectively). 3D6 also significantly lowered the amount of aggregated Aβ42 compared to irrelevant IgG2a (Fig. 3f, *p* = 0.041), and cleared aggregated Aβ40 in 50 % of mice treated with this antibody (Fig. 3e). c*h*Gantenerumab and mC2 did not induce significant changes to aggregated Aβ levels.

### Inflammatory changes

**Fig. 4:**
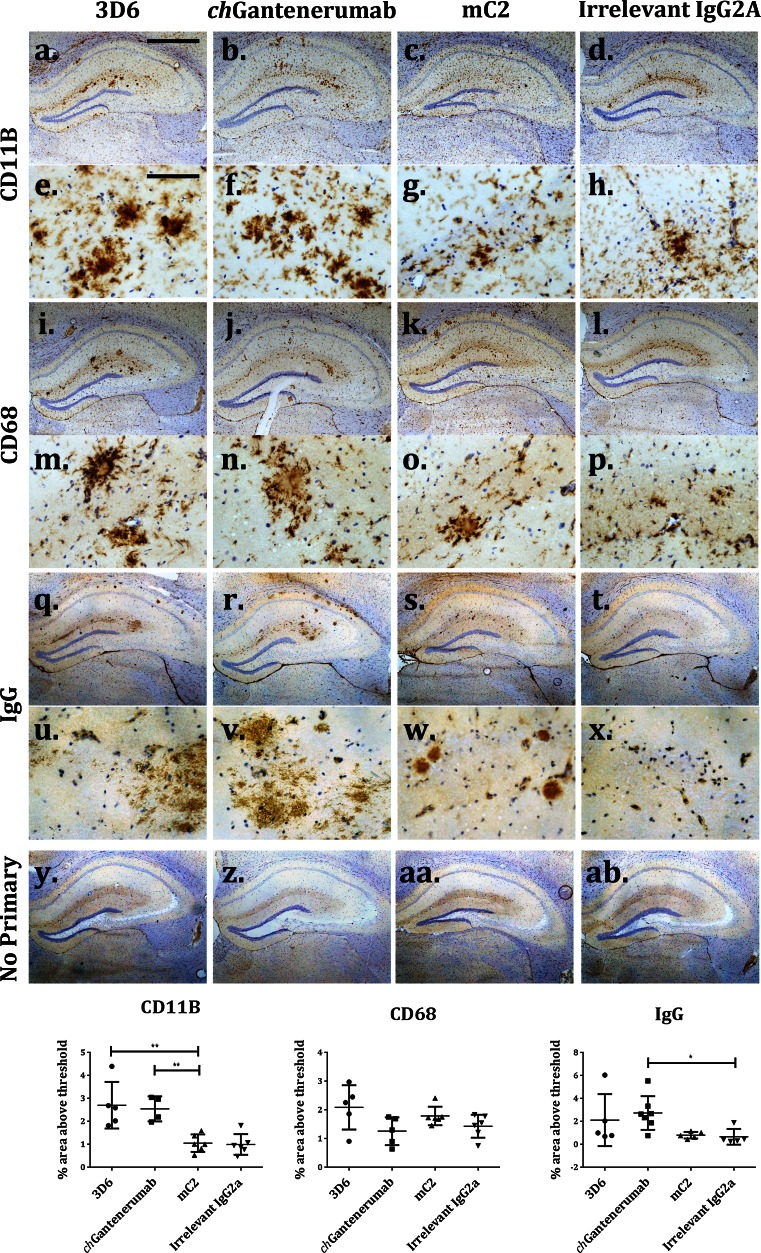
Expression of microglial activation markers after antibody injection. Expression of microglial markers CD11B and CD68 and IgG distribution in the hippocampus 7 days after injection of anti-Aβ antibodies. **a–h** Representative images of CD11B expression. **i–p** Representative images of CD68. **q–x** Representative images of IgG distribution. **y–ab** No primary control sections. Pictures are taken with a 5× objective and 40× objective, *scale bars* 100 and 800 µm, respectively. Staining was quantified as area above threshold of staining and analysed by one-way ANOVA and Tukey post hoc test (*n* = 6/7). 3D6 induced a significant increase in the expression of CD11B, compared to injection with mC2 and irrelevant IgG2a (Fig. 4a–h, *p* = 0.0023 and *p* = 0.0017, respectively). *ch*Gantenerumab also induced CD11B upregulation in comparison to mC2 and irrelevant IgG2a (*p* = 0.0093 and *p* = 0.007, respectively). *ch*Gantenerumab has significantly higher levels of IgG than control injected animals (*p* = 0.011)

Neuroinflammation is thought to be a main cause of side effects in patients treated with anti-Aβ antibodies, therefore, we compared the ability of each of the antibody to induce inflammation after intracerebral injection. We first analysed changes in microglial phenotype by immunohistochemistry. Injection of 3D6 induced a significant increase in the expression of microglial marker CD11B, compared to injection with mC2 and irrelevant IgG2a (Fig. 4a–h, *p* = 0.0023 and *p* = 0.0017, respectively). *ch*Gantenerumab also induced CD11B upregulation in comparison to mC2 and irrelevant IgG2a (*p* = 0.0093 and *p* = 0.007, respectively). We have previously shown CD11B to be upregulated after IgG immune complex formation in the brain [27], suggesting that the increase in CD11B may be due to FcγR binding and subsequent activation of microglia. To assess phagocytic activity, we analysed expression levels of CD68 and detected an increased CD68 expression on microglia in 3D6 injected animals, but not in chGantenerumab injected animals, although the increase was not significantly different (Fig. 4i–p, *p* = 0.08). Figure 4q–x shows staining for mouse IgG, and there is evidence of target engagement by all three of the anti-Aβ antibodies following intracranial injection, as plaques are positive for IgG. Quantification shows that *ch*Gantenerumab has significantly higher levels of IgG than control injected animals (Fig. 4 q–x, *p* = 0.011). Unlike in vitro binding assays, there was no evidence of the *ch*Gantenerumab binding to neurons.

**Fig. 5:**
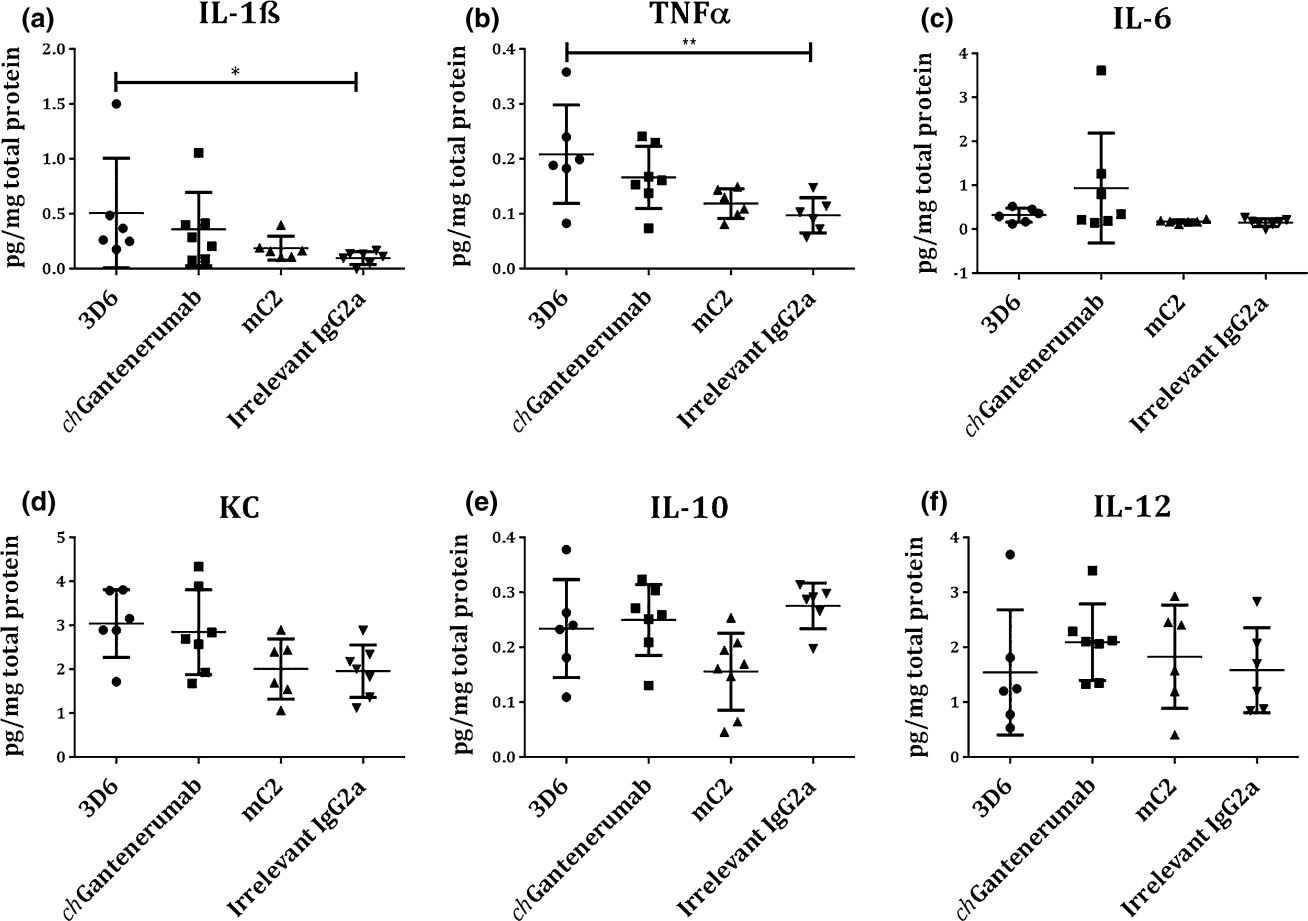
Cytokine levels following intracranial injection of anti-Aβ mAbs. Cytokine levels were measured in homogenate made from hippocampal punches taken from Tg2576 mice injected with antibodies. Peptide levels were measured by multiplex ELISA and normalised to total protein concentration. **a–f** Cytokine levels in hippocampal punches expressed as pg.mg total protein. Injection of 3D6 leads to significantly increased levels of pro-inflammatory cytokines TNFα and IL-1β compared to IgG2a control (**a** *p* = 0.0042, **b** *p* = 0.0262). Data were analysed by one-way ANOVA and Tukey post hoc test (*n* = 6/7), and presented as mean ± SD

We showed that in vitro, all three Aβ antibodies engage FcγRs resulting in macrophage activation and TNFα secretion. Therefore, we next investigated the neuroinflammatory potential in vivo. Injection of 3D6 leads to significantly increased levels of pro-inflammatory cytokines TNFα and IL-1β compared to IgG2a control (Fig. 5a, b, *p* = 0.0042, *p* = 0.0262). Increased levels of KC/GRO in 3D6 injected animals were observed, although this did not reach significance (Fig. 5d, *p* = 0.10). Injection of *ch*Gantenerumab also results in increased neuroinflammation, while mC2 did not affect any cytokine levels measured. These observations suggest that high affinity IgG2a anti-Aβ antibodies reduce Aβ load but this is associated with increased neuroinflammation.

## Discussion

There have been a number of high profile clinical disappointments for anti-Aβ immunotherapy, with Bapineuzumab, Solanezumab, Crenezumab and Gantenerumab all failing to show disease-modifying effects in clinical trials. Differences in: epitope and affinities for Aβ, FcγR affinity and the pre-clinical models used for characterisation make direct comparison difficult. Therefore, to better understand the underlying biological mechanisms, we compared three highly characterised clinical candidates, Bapineuzumab, Gantenerumab and Crenezumab for their ability to clear plaques and induce neuroinflammation in an experimental model of Alzheimer’s disease (Tg2576). We generated murine, recombinant versions of Bapineuzumab (3D6), Crenezumab (mC2) and a mouse/human chimeric version of Gantenerumab (*ch*Gantenerumab). These antibodies were produced using the same murine IgG2a constant regions, and were injected intracranially into 18-month Tg2576 mice. The murine IgG2a constant region is most similar to human IgG1 and has a strong ability to activate human FcγRs and induce inflammation [4]. We are not aware of previous studies that directly compare murinised versions of Bapineuzumab, Crenezumab and Gantenerumab for their ability to clear plaques and induce inflammation.

We show that all IgG2a antibodies bind to recombinant immobilised peptide and plaques present in the Tg2576 brain tissue sections, with 3D6 displaying the highest relative affinity to Aβ. The antibody *ch*Gantenerumab also bound with high affinity to immobilised Aβ peptide, but showed strong binding to neurons in cryosections of both transgenic and wild-type animals. mC2 was able to bind Aβ peptide in vitro and plaques in vivo, but the relative affinity was 100-fold weaker than for 3D6. These observations are relevant for biological function, as 3D6, which had the strongest affinity for Aβ plaques in our model, was the only antibody to significantly reduce Aβ levels in vivo. Due to the potential of antigenic masking to affect the results from immunohistochemistry and MSD assay, we used Congo red staining and demonstrate that plaque numbers are reduced following 3D6 treatment. 3D6 injection also induced the activation of microglia as measured by their increase in CD11B expression and increased levels of the pro-inflammatory cytokines, IL-1β and TNFα. One of the mechanisms of antibody mediated plaque clearance is through activation of microglia FcγRs and phagocytosis [30, 31]. Crosslinking of FcγRs by immune complexes leads to switching to an “M2b” phenotype [18], characterised by the production of reactive oxygen species and pro-inflammatory cytokines, including IL-1β and TNFα. The antibody subclass we used for this experiment, mouse IgG2a, is a potent inducer of inflammation due to its high affinity for activating receptors [5]. It has previously been shown that the IgG2a version of the anti-pyroglutamate mE8 is better at clearing plaques than the IgG1 version (with lower FcγR affinity), implying that FcγRs are involved in the clearance of plaques [10]. Gantenerumab did not significantly reduce the levels of Aβ, but detection of mouse IgG bound to plaques provides clear evidence for target engagement. Like 3D6, increased expression of CD11B was observed but minimal change in CD68 expression levels suggest less efficient phagocytosis following injection of *ch*Gantenerumab. As all antibodies were generated as IgG2a isotypes, this lack of phagocytosis and plaque clearance is not due to lack of effector function, but rather dependent on the affinity and/or epitope. Bapineuzumab and Gantenerumab bind to residues in the N terminus of Aβ, crystal structures of Bapineuzumab in complex with Aβ show that it binds to amino acids 1–5 which are in helical conformation [17]. Gantenerumab, in contrast, binds to a conformational epitope which also includes residues in the mid-domain [3]. c*h*Gantenerumab has a lower EC50 in our Aβ1-40 binding ELISA, when compared to murine 3D6 (0.34 vs 0.17 pM, respectively) and therefore, clearance of plaques may require more time—this is supported by the changes in microglial phenotype and cytokine production. The antibody mC2 was unable to clear Aβ plaques 7 days post-injection, but unlike *ch*Gantenerumab, this antibody did not induce any detectable changes in microglial phenotype or cytokine levels, despite having similar effector function. Immunoreactivity for mouse IgG strongly implies that the lack of clearance is due to poor plaque engagement, as IgG levels were not different from control IgG2a antibody. mC2 binds to a mid-domain epitope (AA 16–24) and studies of other antibodies that bind this region such as, m266 (Solanezumab), suggest that this epitope is inaccessible when Aβ is in aggregated forms and this prevents antibodies like m266 from binding to and clearing plaques [11]. This is supported by binding to formic acid-treated sections, which shows that mC2 requires the solubilisation of plaques with formic acid to bind. Our results demonstrate that targeting the N terminus of Aβ is more effective for plaque clearance. The ability of Crenezumab and Solanezumab to engage human Aβ in AD cases has previously been questioned, and cross-reactivity for a number of antigens has been reported [28]. We, however, found that mC2 was able to bind to Aβ in immunohistochemistry of human AD tissue. Antibodies that are unable to bind to Aβ plaque without formic acid antigen retrieval may not be able to engage plaques in the brains of AD patients. The inability to engage plaques strongly may prevent the removal of aggregated Aβ species from the brain following immunotherapy.

AD patients treated with Bapineuzumab or Gantenerumab have developed dose-limiting vascular side effects (ARIAs), but the underlying mechanisms are not completely understood. There is evidence that activation of microglia, and/or perivascular macrophages through FcγRs and the subsequent neuroinflammation may be partly responsible for these side effects. It has been shown that reduced binding to FcγR can decrease the incidence of vascular damage in mice [6, 13, 29]. In this study, we provide further experimental evidence that antibody engagement with activating FcγRs generates a pro-inflammatory response in the brain, as IgG2a versions of 3D6 and *ch*Gantenerumab both resulted in microglial activation and elevated cytokine production. The clinical candidate Crenezumab is built on a hIgG4 backbone, which has significantly reduced FcγR affinity compared to hIgG1. This was aimed to reduce the inflammatory response to immunotherapy and therefore reduce the associated side effects, and indeed ARIAs were not reported in patients treated with 10x the maximal dose of Bapineuzumab [1]. Our study provides an alternative explanation for the lack of side effects: the lack of plaque engagement by the antibody. We show that intracranial injection of an IgG2a murine version of Crenezumab-mC2 failed to clear plaques or induce a pro-inflammatory response. These observations suggest that inflammation related to immunotherapy is not just dependant on the ability of the antibody to engage FcγRs but also on the epitope and ability of the antibody to engage plaques.

3D6 and mC2 are not exactly the same as the final clinical candidates; Bapineuzumab and Crenezumab. This could mean there are slight differences in the binding affinity which could influence the rate of Aβ clearance and inflammation; therefore, extrapolating these data should be done with caution. However, the clinical antibodies share the same epitope and are incredibly similar in sequence, and therefore the response is likely to be similar. Intra-cranial injection is a useful technique allowing rapid and cost-efficient characterisation of the response to an antibody, however, there are some limitations to this approach. To limit injection-mediated tissue damage, we use fine pulled glass capillaries to inject into the brain. However, even with caution any injection will cause some tissue damage, which may influence the subsequent immune response. Patients are not injected intracranially with these antibodies, and therefore, injecting directly into the brain is less relevant to the clinic than giving a systemic dose of the antibodies. One factor to consider is that the localised concentration of IgG following central injection will be higher than after systemic treatment, which could therefore influence the neuroinflammatory response. To fully characterise the differences between these antibodies, a systemic trial is needed which would also allow the measurement of cognitive changes. Previous studies have shown that there is variability in the response between different transgenic APP mouse models, in particular extensively aged mice, after treatment with anti-Aβ antibodies [10, 33]. Therefore, further studies would be required to confirm the findings presented in this manuscript in different models. Furthermore, studies in transgenic APP mice have not always translated into successful clinical trials in humans; therefore, the response to the different anti-Aβ antibodies may differ in human AD patients.

We believe the results presented in the current manuscript can inform the development of optimised clinical antibodies. Treatment with 3D6 is effective at clearing plaques, however, part of this response causes increased neuroinflammation. Optimised immunotherapy for AD ideally separates phagocytosis and neuroinflammation, which would allow clearance of Aβ without the induction of detrimental pro-inflammatory cytokine release. To achieve this, better understanding of the receptors involved in this processes is essential. We know that a range of different FcγRs is expressed in the brains of AD patients and mouse models [8, 14, 21], but the physiological role of these receptors in the brain and their relative contribution to neuroinflammation and plaque clearance remain poorly understood. 3D6 has strong target binding and a high risk of causing vascular side effects and inflammation, therefore, to minimise toxicity reduction of its effector function will likely be beneficial. In contrast, mC2 with lower target binding might need stronger FcγR binding to increase efficacy. Based on our results, we conclude that reduction of the effector function of Crenezumab did not have the expected beneficial effects, and the lack of clinical efficacy may be due to its lower plaque engagement. It is clear that we do not fully understand the role that FcγRs play in the CNS (see recent review [14], as there is mounting evidence that these receptors play a specific role in the pathology of dementia. In humans, six FcγRs have been identified (activatory receptors, FcγRI, IIa, IIc, IIIa, an inhibitory receptor FcγRIIb and a GPI-linked decoy FcγRIIIb) and the ratio of activatory: inhibitory expression determines the activation threshold of effector cells [19]. Different receptors may play different roles in the clearance of Aβ or in the induction of side effects. Effector function of antibodies is critical to cancer immunotherapy, and much progress has been made in antibody engineering to develop therapeutic antibodies with improved binding to a particular receptor [7]. Better understanding of the role of specific FcγRs in immunotherapy may facilitate the modification of anti-Aβ immunotherapies by antibody engineering to maximise plaque clearance and reduce side effects.
